# Optical vacuum cleaner by optomechanical manipulation of nanoparticles using nanostructured mesoscale dielectric cuboid

**DOI:** 10.1038/s41598-019-49277-9

**Published:** 2019-09-04

**Authors:** Igor V. Minin, Oleg V. Minin, Yinghui Cao, Zhenyu Liu, Yuri E. Geints, Alina Karabchevsky

**Affiliations:** 1 Tomsk Polytechnic University, 36 Lenin Avenue, Tomsk, 634050 Russia; 2 Tomsk State University, 30 Lenin Avenue, Tomsk, 634050 Russia; 3 College of Computer Science and Technology, Jilin University, 2699 Qianjin Street, Changchun, 130012 China; 4 Changchun Institute of Optics, Fine Mechanics and Physics, 3888 East Nanhu Road, Changchun, 130033 China; 5 V.E. Zuev Institute of Atmospheric Optics SB RAS, Zuev square 1, Tomsk, 634021 Russia; 6 School of Electrical and Computer Engineering, Ben-Gurion University of the Negev, Beer-Sheva, 8410501 Israel

**Keywords:** Optical physics, Optical physics

## Abstract

Here, we propose the concept of an “optical vacuum cleaner” for optomechanical manipulation of nanoparticles. We utilize a dielectric cuboid to generate an optical gradient force exerted on the nanoparticles for particle’s hovering and trapping. We show that the permittivity contrast between the particle and the nanohole leads to the deep subwavelength light confinement and enhancement at the opening of the nanohole located at the shadow surface of the particle. The proposed “optical vacuum cleaner” can be utilized in optomechanical manipulations on particles such as noble metal nanoparticles adsorbed on surfaces or controlling the particles taking part in cellular uptake.

## Introduction

Overcoming the diffraction limit and localizing light on the subwavelength scale have a remarkable impact in the field of optics, with applications toward imaging, communication, and sensing systems. For this, a wide range of optical components for subwavelength light localization have been proposed. Tight focusing of light beyond the diffraction limit such as 3D diffractive optics elements^1^, all-dielectric photonic crystal-based (PhC) lenses^2–6^ and plasmonic lenses with metallic/dielectric nanoslit^7–9^ have been investigated. However, the fabrication of PhC and plasmonic devices rely on fabrication state-of-the-art techniques such as electron beam lithography and reactive ion etching^10,11^. However, those techniques are overpriced not suitable for large-scale mass production. Other configurations such as nanohole-structured dielectric objects to explore the effects of total light scattering of deep holes drilled in spherical particles with refractive index near n~1.05 were studied in^12^. However, the authors showed that the influence of the holes is negligible. Graded PhC lenses with varying-sized air holes were studied as well^13,14^. It has been reported that by means of a graded PhC lens, the focused light beam with a Full Width at Half Maximum (FWHM) of the beam-width is about λ/75 could be achieved^14^. To control the overlap in spectrum of the Mie-type resonant modes and the scattering patterns of particles, a dielectric nanorod with a hole along its axis was recently proposed^15^.

Dielectric mesoscale microparticles, named as “photonic nanojet” phenomenon (PNJ), are also used for light focusing at the subwavelength regime^16–20^. For micron-sized spheres made of dielectric materials such as glass and plastic with refractive index less than two, the minimum beam-width of produced PNJ is about λ/3^18^. To reduce the PNJ beam-width, structured microspheres with concentric rings on the surface were investigated^21^. Recently, it was reported that one can reduce the size of the focal spot of dielectric mesoscale sphere due to the nanohole located in its shadow surface. The nanohole located in its shadow surface improved in turn the spatial resolution up to λ/40. The improvement is beyond the solid immersion diffraction limit (λ/2n)^22^.

According to geometrical optics approximation and the Snell’s law, for a spherical particle with refractive index of n = 2, a light focal spot is located at the shadow surface of the microsphere^19,20^. Interestingly, numerical simulations show that for particles with sizes comparable to the wavelength (R~ λ), and even with nonspherical geometrical shape (e.g., cubic particles), the focal spots are still located near their shadow surface^19,20,23^. In addition, for cubic and spherical particles which have the same refractive index and same dimensions of about one wavelength, the FWHM beam-width of the photonic jet that generated by the cubic particle (equilateral cuboid) is smaller than that of the spherical one^20^, thus we choose to investigate light focusing properties and optical forces of the nanohole structured cuboid.

Here, we propose the concept of “optical vacuum cleaner” which is based on the nanohole structured dielectric cuboid for particle’s manipulationat nanoscale. To introduce the concept, we first numerically simulate the nanohole structured dielectric cuboid and study its light focusing properties. Second, we analyze the multipole scattering efficiencies of the simulated particle and finally we calculate the optical forces exerted on an object, here a gold nanoparticle which is close to the shadow surface of the simulated cuboid.

## Light Focusing Properties of Dielectric Cuboid

**Figure 1:**
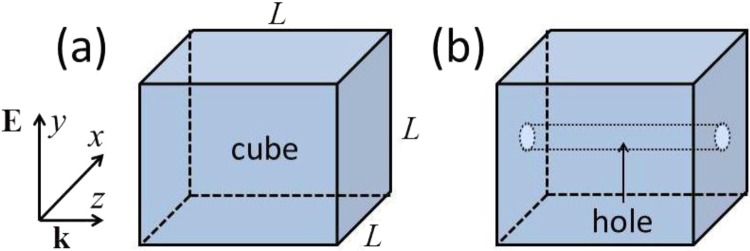
Schematic diagram for (**a**) the bulk cuboid without a hole and (**b**) the nanohole structured cuboid.

In this section, the light focusing properties of dielectric cuboid with edge of *L* = *λ* are numerically investigated. Schematic diagrams for the simulated cuboid, including a bulk cuboid (cube without hole) and a nanohole structured cuboid, are plotted in Fig. 1a,b respectively.

### Cuboid without nanohole

We model the dielectric cuboid using the the Finite Element method (FEM) software COMSOL Multiphysics. In the simulation, the incident light was assumed as a plane wave, linearly polarized along the y-axis and propagating along the *z*-axis as shown in Fig. 1. Table 1 shows the focal spot volume *V*, calculated as a volume integral over the FWHM focal spot of the dielectric cuboid (see Fig. S1 from Supplementary Materials). The last column of Table 1 shows the enhanced intensity defined as *I*_max_/*I*_0_ which is maximal intensity *I*_max_ normalized to the incident light intensity *I*_0_.

**Table 1:** Light focusing properties of dilectric cuboids, including the focal volume *V* and the maximum light intensity *I*_*max*_.

n	*V/λ* ^3^	*I*_*max*_ */I*_*0*_
1.6	0.0505	14.4
1.8	0.0234	16.3
2.0	0.0124	19.5
2.2	0.0150	12.5

The cuboids have the size of one wavelength (L = λ) but with referactive index of n = 1.6, 1.8, 2.0, and 2.2, respectively.

The subwavelength field enhancement of these mesoscale dielectric particles is a near-field optical effect, which couples fields between the evanescent waves and the propagating waves^19^. In addition, for particles with relative refractive index *n* < 2, the maximal field intensity is located beyond the particle boundaries. This contradicts the geometrical optics principles. While increasing the particle refractive index *n*, the focal spot moves toward the shadow surface of the cube. When *n* approaches 2, the focal spot is located at the shadow surface of the particle, and the focusing volume *V* reaches its minimum value of 0.0124 λ^3^. For *n* > 2.2, the focal spot moves into the cuboid, and only a small part of the high-intensity region is located outside the cuboid. Here, we consider a cuboid of *n* = 2, which can be made of conventional materials commercially available in optical frequencies^24^.

### Cuboid structured with nanohole of cylindrical shape

**Figure 2:**
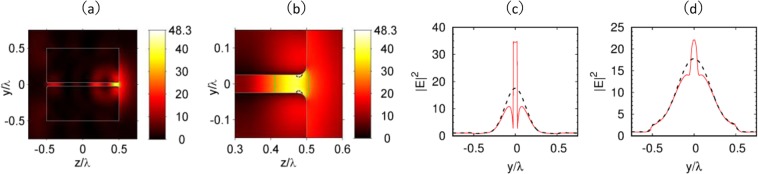
Optical intensity (E^2^) of the proposed nanohole structured cubic particle in (**a**) polarization plane (z-y plane), (**b**) around the focal spot. The refractive index and size of the simulated cuboid are set to be *n* = 2 and *L* = λ, and diameter of the nanohole is *d*_*h*_ = λ/20. The Full width at Half Maximum (FWHM) and focal spot areas are indicated by green solid lines. To demonstrate the extreme field localization inside the nanohole, contour lines at intensity level of 0.85*I*_max_ are plotted with dashed lines in (**b**). (**c**,**d**) The red solid lines represent light intensity profiles along the cut-line of (**c**) *x* = 0 and *z* = 0.45 λ, (**d**) *x* = 0 and *z* = 0.51 λ, respectively. The dashed lines represent light intensity profiles along the two cut-lines of solid cuboid without a hole.

Figure 2a,b illustrate the 2D-distribution of light intensity (*I* = |**E**|^2^) of a nanohole-structured dielectric cuboid, with refractive index *n* = 2, hole diameter *d*_h_ = λ/20 and the corner radius of the hole opening *r*_c_ = λ/20. We note, that while decreasing the corner radius *r*_c_, the maximal field intensity *I*_max_ inside the focal spot increases as well due to the increase in the curvature of the nanohole opening. In fact, our simulation shows that dividing the corner radius r_c_ by two corresponds to a 13% increase of the maximum intensity.

From Fig. 2a,b, one can see that the electric field 3D-localization effect inside and near the hole boundary is similar to the electric field 3D-localization in a slot waveguide^25,26^. In such a waveguide structure, the slot is of lower refractive index than the cladding and concentrates the electromagnetic energy inside the waveguide. In addition, the observed effect resembles the field enhancement in spherical particle with a nanohole^22^. Compared to the slot waveguide^25^, the proposed nanostructured dielectric cuboid has lower refractive index, and the nanohole is completely surrounded by the dielectric medium, providing electrical field confinement inside the hole due to the refractive index contrast between the hole and the dielectric cuboid.

Figure 2c,d show the light intensity along the cut-line of *x* = 0, *z* = 0.45λ, and *x* = 0, *z* = 0.51λ respectively. The shadow surface of the cube is at *z* = 0.5λ, thus the two cut-lines are −0.05λ and 0.01λ from the shadow surface respectively. As follows from Fig. 2c,d, the optical field is strongly confined in the nanohole where it exhibits large discontinuity at the hole boundaries. Similar to the spherical particle^22^, the optical field is localized mainly in a small region near the shadow surface of the cube, while partially occupying the hole. Moreover, numerical simulation shows that, dielectric cuboid with a blind nanohole can be used to produce light spot with a little higher maximum intensity than that produced by cuboid with a penetrating hole similar to spherical particle^22^ as shown in subplots of Fig. S2 from Supplementary Materials.

Considering that the optical gradient force is mainly dictated by the gradient of light intensity around the light spot, we can expect that cuboid with a blind nanohole will produce optical gradient force which is a little stronger than that of cuboid with a penetrating nanohole. We notice that the difference is minor though. This can be explained by the materials properties of dielectric cuboid with a blind nanohole which contains more dielectric material at its center, thus has a higher light focusing capability than the cuboid with a penetrating nanohole. On the other hand, the proposal of cuboid with a blind nanohole is based on the fact that the optical gradient force is mainly distributed around the focal spot, thus one can just drill a nanohole around the focal spot to utilize the optical gradient force. This allows for setting the hole depth not to the entire length of the cubic edge *L*, but only to a part of it, e.g., a blind hole with an entrance at the shadow surface of the cube.

It is worth noting that the nanohole-structured dielectric mesoscale particles possess several unique properties. For example, they are capable to produce light beam with high intensity and high optical power in the region of relatively lower refractive index (i.e., air). One cannot achieve this with the conventional PNJ generated from particles of the same sizes^19,20^. This feature allows for highly efficient coupling of electric fields between the nanohole structured particles and surrounding materials, as well as optical components that may contain some optically active material. As an example, this may provide parametric amplification and all-optical switching in integrated photonics^27^. Moreover, as light field is strongly confined in the nanohole, the proposed nanohole-structured dielectric cuboid can be used as near-field probe, or as a compact optical sensing device.

## Multipole Analysis of the Cuboid

**Figure 3:**
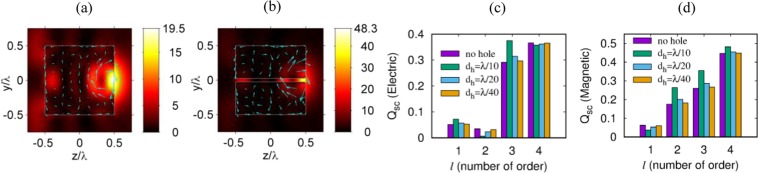
(**a,b**) Optical intensity (*I* = |**E**|^2^) and vectors of the electric polarization (**P** = χε_0_**E**) of the solid and nanohole structured cuboids. (**c,d**) Electric and magnetic multipole scattering efficiencies of the solid and nanohole structured cuboids. The refractive index and size of the simulated cuboids are set to be *n* = 2 and *L* = λ, and diameter of the nanohole is *d*_*h*_ = λ/20. Arrows in the figures (**a**,**b**) represent the polarization vector components *P*_*z*_, and *P*_*y*_ in the polarization plane. The integer *l* in the figures (**c**,**d**) denotes the order of the multipole, which indicates dipole, quadrupole, octupole and hexapole, for *l* = 1 ~ 4, respectively.

Next, we explore the multipole moments excited in the cuboid. In Fig. 3(a,b), the light intensity (*I* = |**E**|^2^) and vectors of the electric polarization (**P** = χε_0_**E**) of the solid and the nanohole structured dielectric cuboid are plotted respectively. The refractive index and size of the simulated cuboids are set to be *n* = 2 and *L* = λ, and diameter of the nano hole is *d*_*h*_ = λ/20 as introduced in Section 2. Figure 3(a,b) show that electric dipole and high order multipoles are excited in the cuboid. To clarify the contributions of different multipole moments, we use the multipole decomposition method^28^. To understand the scattering effect from the cuboid we calculate the scattering efficiencies of the electric and magnetic dipoles, quadrupoles, hexapoles, and octupoles presented in Fig. 3c,d. We note significant octupolar and hexapolar contributions to the scattering cross-section while the magnetic multipoles contribution is comparable to the contribution of electric multipoles. From Fig. 3c,d, we find that if a nanometer hole is arranged inside the cubic particle, the contribution of the magnetic dipole slightly decreases, while contribution of the electric dipole is slightly enhanced. The contribution of electric and magnetic quadrupoles demonstrates opposite behavior: with the increase of the nanohole diameter, the electric quadrupole scattering efficiency decreases, while the magnetic counterpart increases. The electric and magnetic hexapole moments are more sensitive to the presence of a hole and increase simultaneously their contribution to the total scattered field when the cuboid is perforated and there is a minor change in octupole moments contribution.

So, at hole diameter of λ/10, the magnetic dipole moment decreases by about 1.3 times, while the electric moment increases by 30%. The electric quadrupole moment decreases by 1.5 times, and the magnetic moment increases by 1.3 times. Hexapole electric and magnetic moment increases by 25%. Octupole moments change slightly. This is due to the week of the wave interference involved in the confinement and guiding mechanisms in analogy with results reported in ref.^25^. The excitation of high-order multipoles corresponds to a more inhomogeneous distribution of fields inside a dielectric particle. Therefore, high-order multipoles are more sensitive to changes in the internal structure of particles. Therefore, the observed effects are caused by the redistribution of the fields inside the particle in the presence of a hole.

Considering the above discussions for the proposed cuboid we notice that the magnetic octupole moment is dominant. In other words, a cuboid of wavelength size is more “magnetic” than “electric” with regards to its optical properties. In addition, for all cases the contributions of magnetic quadrupole moments are several times higher than that of the electric quadrupole moments. This behavior shows a surprising feature of Mie scattering: the mesoscale cubic particles induces such anomalous scattering effect. Namely, the excitation of optical resonances with inverted hierarchy (it’s unclear to me what this means) when the quadrupolar resonances are more pronounced compared to the dipolar resonances.

**Figure 4:**
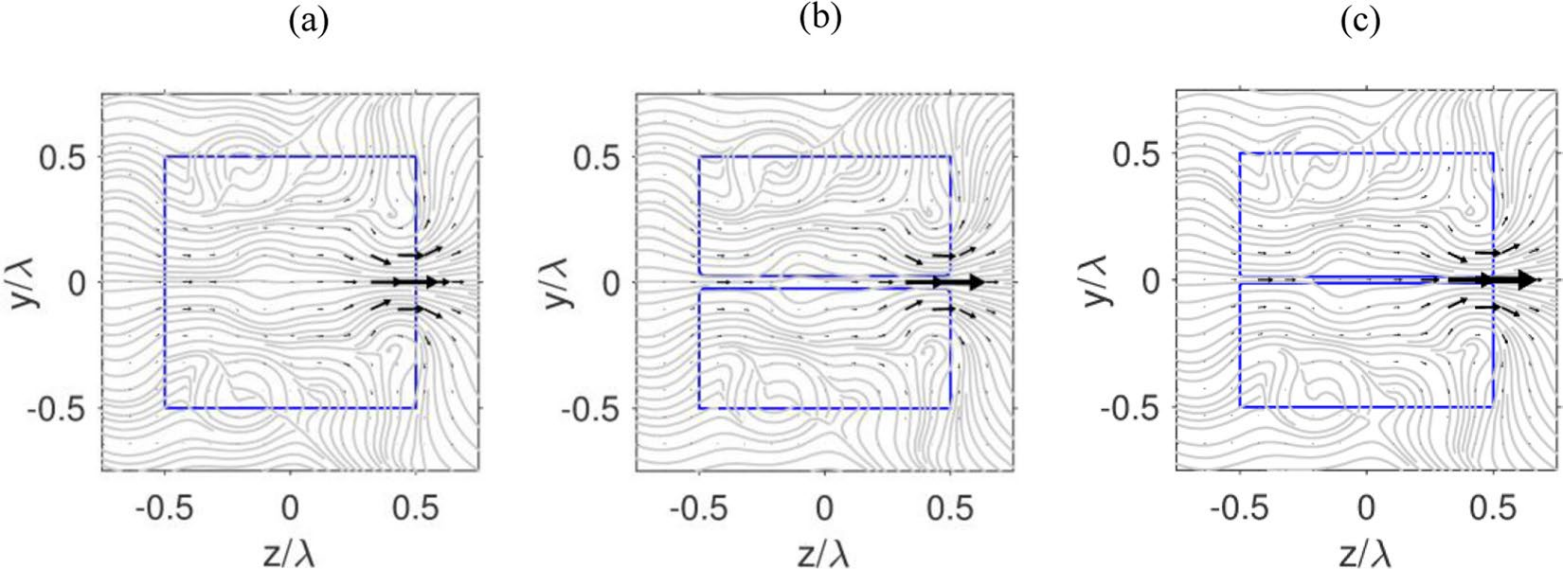
Poynting vectors of (**a**) a solid cuboid without nanohole, (**b**) a nanohole structured cuboid with hole diameter *d*_*h*_ = λ/20, and (**c**) *d*_*h*_ = λ/40, in the z-y plane (polarization plane). The refractive index and size of the simulated cuboids are set to be *n* = 2 and *L* = λ.

Now, we consider the energy fluxes in the bulk cuboid (without a nanohole structuring) and the nanohole structured cuboid presented in Fig. 4a,c, which show the physical mechanism of the optical field localization near the nanohole. One can see that the Poynting vector distributions include singular points. The number, types, and positions of the Poynting vector depend on the hole dimensions. The vortices observed in the energy flows are the foci in the phase space^29^. Two distinct energy flows are clearly observed in a cuboid without the nanohole: (a) the central energy flow produces the focusing region near the shadow particle surface, and (b) the peripheral energy flow that is directed toward the corners of the cube creates side scattering lobes in the near-field. The introduction of the hole-like strucutre reduces the peripheral energy flow and also leads to the increase of the energy localization in the shadow particle surface in the region of the nanohole. In this case, the decrease in the hole diameter leads to the increase in the z-component of the Poynting vector.

## Optical Vacuum Cleaner Concept

**Figure 5:**
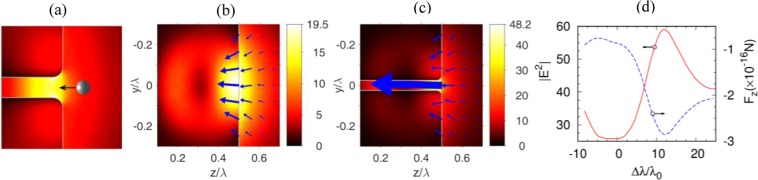
(**a**) Schematic diagram for the “optical vacuum cleaner”, where a nanoparticle is pulled by the optical force, and moves towards the nanohole structured dielectric cuboid. (**b**,**c**) Light intensity (|E|^2^) and optical force distributions for (**b**) solid cuboid without a hole, (**c**) cuboid with a 20 nm hole. The refractive index and size of the simulated cuboids are set to be *n* = 2 and *L* = λ. (**d**) Optical force and light intensity vs illumination wavelength, at the opening of the nanohole. The optical force is assumed to be exerted on a gold nanosphere with the radius of *d* = 15 nm and complex dielectric permittivity ε_p_ = −9.421 + 1.504 i at λ = 600 nm^35^, and calculated using the dipole approximation. The arrows in blue color represent the optical gradient force.

The multipoles analysis of the structure, presented in the previous section, reveals an interesting effect that we discuss here. As it can be seen from Fig. 4b, in a nanohole structured cuboid, the energy flow penetrates into the nanohole area at the shadow surface. This inward energy flow behavior can lead to the interesting practical applications such as an “optical vacuum cleaner” device. The key idea is to use our specially designed nanohole structured cube for optical momentum redirection and achieving the desired mechanical effects on a nanoscale target object as shown in Fig. 5.

It is known, that the principle of operation of an “optical trap”^30–33^ relies on some mechanical action of the electromagnetic field applied on an object. Acting on an object, the optical force depends on light intensity and field spatial gradient at the location of the object^30,31^. Bearing this in mind, one can define two types of optical forces: one is the scattering force **F**_*p*_ that is directed along the Poynting vector, and another is the gradient force **F**_*g*_, which is the Lorentz force of the electromagnetic field acting on the dipoles induced in the object. If a particle with higher refractive index than the surrounding medium is placed in an inhomogeneous light field, scattering force **F**_*p*_ and gradient force **F**_*g*_ make particle to move toward the lens focus - the region with higher light intensity. It is important to stress, that the scattering force is nonconservative force and therefore is leads to the unrestricted particle motion. The gradient force however, is responsible for the particle trapping^31,32^.

Figure 5a shows a schematic diagram for the “optical vacuum cleaner”, where a nanoparticle is pulled by the optical force, and moves towards the nanohole. A cuboid particle has advantages over the spherical particle^22^ due to its flat surfaces. Light intensity (|E|^2^) and optical force distributions of a dielectric cuboid without a hole, and a cuboid with a λ/20 hole are shown in Fig. 5b,c, respectively. The refractive index and edge length of the cubic particle are set as *n* = 2 and *L* = λ. Comparing with Fig. 5b,c, we can find that, at the opening of the nanohole, the optical force is considerably enhanced because of the field confinement within the nanohole. Figure 5c shows that the main optical trapping region is located in the high-intensity area near the hole opening. In addition, for a nanohole structued cuboid with fixed structure parameters, Fig. 5d shows its optical force and light intensity at the opening of the hole, versus varying illumination wavelength. In Fig. 5d we note that the maximal values of the optical forces can be obtained by increasing the wavelength by about 12%. This is because while the illuminating wavelength decreases, then the effective size of the dielectric particle^20^ increases, thus the position of the light focusing spot shifts from the inward of the cuboid towards the shadow surface of the cuboid.

**Figure 6:**
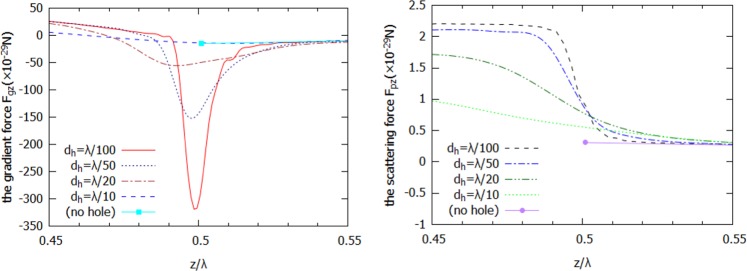
*Z*-components of (**a**) optical gradient force *F*_*gz*_ and (**b**) optical pressure (scattering force *F*_*pz*_) for cuboid with *n* = 2 and different hole opening diameters. The shadow surface of cuboid is located at *z* = 0.5λ, and the probe particle is assumed to be gold nanosphere with diameter of *d* = 5 nm. The incident light is a plane wave with amplitude of *E*_0_ = 1 V/m and wavelength of λ = 600 nm.

Finally, we have calculated the optical forces acting on a gold nanoparticle placed within a hole at the plane of shadow surface of cuboid, as shown in Fig. 6, for both the optical gradient and the scattering force components. We assume that the incident light has the electric field intensity of *E*_0_ = 1 V/m, and the probe particle is a gold nanosphere^34^ with the diameter of 5 nm. The edge length of the dielectric cuboid with hole is set as *L* = λ. The wavelength of the incident light is 600 nm. As shown in Fig. 6, z-component of the gradient force **F**_*g*_ becomes negative at the hole opening, pulls the probe particle through the hole, toward the regions with higher optical intensity. In the above cases, the optical scattering force **F**_*p*_ is much smaller than an optical gradient force **F**_*g*_, thus we can consider the gradient force **F**_*g*_ but, ignoring the scattering force **F**_*p*_^35^. When decreasing the hole diameter, the minimum of an optical gradient force shifts closer to the shadow surface of the particle (towards the entrance of the hole), and the gradient force is considerably enhanced and exceeds as much as more than 20 times in its value for a cube without a hole.

To illustrate the trapping ability, we numerically solved the equation of motion (2). The solution of this equation is carried out numerically in the 3D-domain. Optical forces are calculated and updated each time as the particle position changes. The computations of the field vectors are carried out using FDTD technique (Lumerical FDTD software package) with an adaptive time step. A 15 nm gold sphere is injected inside the trapping region at the point with the coordinates (0, 0, λ/4) and then it begins to move towards the trapping region under the action of net mechanical force.

**Figure 7:**
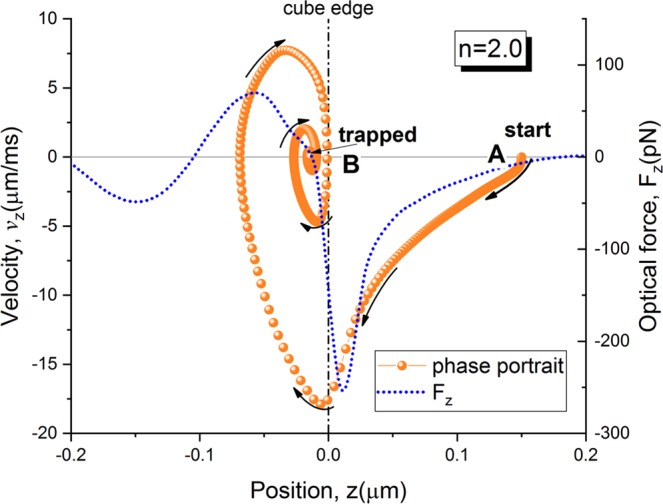
Phase portrait of 15 nm gold nanoparticle motion in the vacuum cleaner optical trap. (**A** and **B**) are initial and final points of the trapping trajectory under 600 nm laser illumination. Shown also is *z*-component of the net optical force, *F*_*z*_, within the trapping region.

Figure 7 demonstrates the phase portrait of the nanoparticle motion within the proposed optical vacuum cleaner trap when exposed to focused laser radiation (NA = 0.5) with λ = 600 nm and the power of 10 W. Note the phase portrait is a trajectory showing the dependence of particle velocity component, namely *v*_*z*_ = *dz*/*dt*, on the corresponding position coordinate, namely *z*. As can be seen, in the starting point “**A**”, the particle begins to move because of the nonzero axial intensity gradient directed toward the cuboid. The uncompensated gravity force along the *y*-axis causes the simultaneous particle motion in the vertical direction. After a few milliseconds, the particle relaxes to the final point “**B**” of its trajectory located approximately 20 nm inside cube hole opening. At this point *z*-movement of gold particle completely stops while it continues oscillating in the *xy*-plane within the hole (not shown).

Thus, the manipulation of nanoparticles by the nanohole structured cuboid could be controlled by changing its structure and material parameters. The proposed “optical vacuum cleaner” may also allow for developing new nanoparticle manipulation method by choosing the shape of the hole, for example, a toroidal shape.

## Conclusion

To conclude, we presented the design of the “optical vacuum cleaner” which is based on a wavelength-scaled dielectric cuboid with perforated air-filled nanohole of cylindrical shape. We analyzed in details the local optical field enhancement in the nanohole, together with the optical forces acting on a target nanoparticle in vicinity with the cuboid. Numerical simulation shows that light can be confined inside the nanohole of the proposed dielectric cube, even when the hole size is as small as λ/40. The spatial dimensions of the field localization area are determined by the nanohole diameter rather than by the incident wavelength. The proposed nanohole-structured microparticle has an advantage that the spatial region of light confinement and enhancement can be tailored by choosing the proper geometry, shape and size of the nanohole. Similar to the metal screen with hole array^36^, at the opening of the nanohole of the proposed cuboid the confined light spot contains evanescent waves with high spatial frequency components.

Nanoparticles manipulation such as capturing and removal from the surrounding medium is crucial in many practical applications, including healthcare^37^, protective clothing, air cabin filters, indoor air purifiers, and air purification systems^38^. Numerical simulation shows that under the condition of plane wave illumination, the proposed nanohole-structured cubic particle can be used for optical trapping and removal of metal nanoparticles, and has a vast potential in chemical, biomedical research for designing the reconfigurable optical devices by sculpturing and removing gold nanoparticles^39^ and other technological applications comparing with traditional optical methods^40^.

## Methods

In this work, the proposed nanohole structured dielectric cuboids are modeled and simulated by using the Finite Element Method (FEM) based software package COMSOL Multiphysics. The simulation domain consists of the cuboid at the center, and the surrounding medium, as well as the Perfect Matched Layer (PML) for terminating the computational domain. The incident light was set as a plane wave, which is linearly polarized along the y-axis and propagating along the z-axis, at the wavelength λ = 600 nm. To obtain accurate electromagnetic fields inside the nanohole, very fine mesh grids are created around the nanohole interface, with minimum element size of λ/100 and λ/200 for the λ/20-hole and λ/40-hole respectively. To calculate the optical force that exerted on a probe particle of gold nanosphere, experimental data of optical constants of gold^41^ is used, with the complex permittivity of ε_p_ = −9.421 + 1.504 i that is obtained by interpolating at λ = 600 nm. For the the gold nanosphere is smaller than the light wavelength, the optical forces can be calculated in the Rayleigh approximation^32,35^:

1 $$\begin{array}{c}{{\bf{F}}}_{p}={n}_{0}{\alpha }^{2}{k}^{4}/6\pi c\\ {{\bf{F}}}_{g}=\alpha {\varepsilon }_{0}\nabla I\end{array}$$

where **F**_p_ and **F**_g_ is the optical pressure and optical gradient force respectively, and $\alpha =4\pi {R}_{0}^{3}{n}_{0}^{2}({n}_{p}^{2}-{n}_{0}^{2})/({n}_{p}^{2}+2{n}_{0}^{2})$ is dielectric particle polarizability, $I=(c{n}_{0}{{\rm{\varepsilon }}}_{{\rm{0}}}/2){|{\bf{E}}|}^{2}$ denotes light intensity, *k* = 2π/λ is the wavenumber, *n*_0_ is ambient refractive index (air), and ε_0_ stands for vacuum permittivity.

To illustrate the trapping ability, we numerically solved the equation of motion of a small non-deformable body with a mass *m* in Earth gravity field under the action of the external optical force^42,43^. According to the Newton’s law we write:

2 $$m\frac{{d}^{2}{\bf{r}}}{d{t}^{2}}={{\bf{F}}}_{opt}-\gamma \frac{d{\bf{r}}}{dt}+m{\bf{g}}$$

Here, *r* ≡ (*x, y, z*) is the vectorial position of particle gravity center, **F**_*opt*_ = **F**_*p*_ + **F**_*g*_ is net optical force, γ = 6πη*R*_0_ is Stokes coefficient of the friction force for a bead moving in a homogeneous medium with the dynamic viscosity η (in air, η = 1.8·10^−5^ kg/(m·s)), *m***g** = −*mg***e**_*y*_ denotes the gravity force acting in the negative *y*-direction (gravity constant, *g* = 9.8 m/s^2^).

## Supplementary information

How to utilize the optical gradient force by drilling a nanohole around the focal spot
