# Supplementary material for: Optical vacuum cleaner by optomechanical manipulation of nanoparticles using nanostructured mesoscale dielectric cuboid
Source: Sci Rep. 2019 Sep 4;9:12748. doi: 10.1038/s41598-019-49277-9 (PMC6726766; doi:10.1038/s41598-019-49277-9)
Supplement: Supplementary file 1 — How to utilize the optical gradient force by drilling a nanohole around the focal spot [file 41598_2019_49277_MOESM1_ESM.pdf]

Optical vacuum cleaner by optomechanical manipulation of nanoparticles using nanostructured mesoscale dielectric cuboid

Igor V. Minin[1,2]*, Oleg V. Minin[1, 2], Yinghui Cao[3], Zhenyu Liu[4], Yuri E. Geints[5], and Alina Karabchevsky[6*]

[1]Tomsk Polytechnic University, 36 Lenin Avenue, Tomsk 634050, Russia. [2]Tomsk State University, 30 Lenin Avenue, Tomsk 634050, Russia. [3]College of Computer Science and Technology, Jilin University, 2699 Qianjin Street, Changchun 130012, China. [4]Changchun Institute of Optics, Fine Mechanics and Physics, 3888 East Nanhu Road, Changchun 130033, China. [5]V.E. Zuev Institute of Atmospheric Optics SB RAS, Zuev square 1, Tomsk, 634021, Russia. [6]Electrooptics and Photonics Engineering Department, Ben-Gurion University of the Negev, Beer-Sheva 8410501, Israel. Correspondence and requests for materials should be addressed to: Igor V. Minin. (email: prof.minin@gmail.com) and Alina Karabchevsky (email: alinak@bgu.ac.il)

SUPPLEMENTARY MATERIAL

A schematic diagram of the light spot area S for a two-dimensional case (as provided in the manuscript), is shown in the following picture S1:

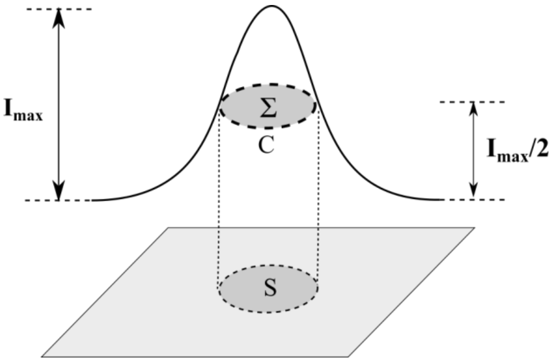

Fig S1. Schematic diagram of the focal spot size for the two-dimensional case, where the focused light spot is composed of a bell-shaped light intensity distribution. The focal spot area is defined as $S = \iint_{\Sigma} dxdy$, where Σ is the surface enclosed by the contour line at half of the maximum light intensity $I =$

$I_{max}/2$. Similarly, for a three-dimensional case, the light spot volume V is defined as $V = \iiint_{\Omega} dxdydz$, where Ω is the region enclosed by the isosurface at half of the maximum light intensity.

Numerical simulation shows that, dielectric cuboid with a blind nanohole can be used to produce light spot with a little higher maximum intensity than that produced by cuboid with a penetrating hole similar to spherical particle [22], please see figures S2. Considering that the optical gradient force is mainly decided by the gradient of light intensity around the light spot, we can expect that cuboid with a blind nanohole will produce optical gradient force which is stronger than that of cuboid with a penetrating nanohole, although the difference is not so big. This effect occurs because the dielectric cuboid with a blind nanohole contains more dielectric material at its center, thus it has higher light focusing capability as compared to the cuboid with a penetrating nanohole. On the other hand, the proposal of cuboid with a blind nanohole is based on the fact that the optical gradient force is mainly distributed around the focal spot, thus one can just drill a nanohole around the focal spot to utilize the optical gradient force.

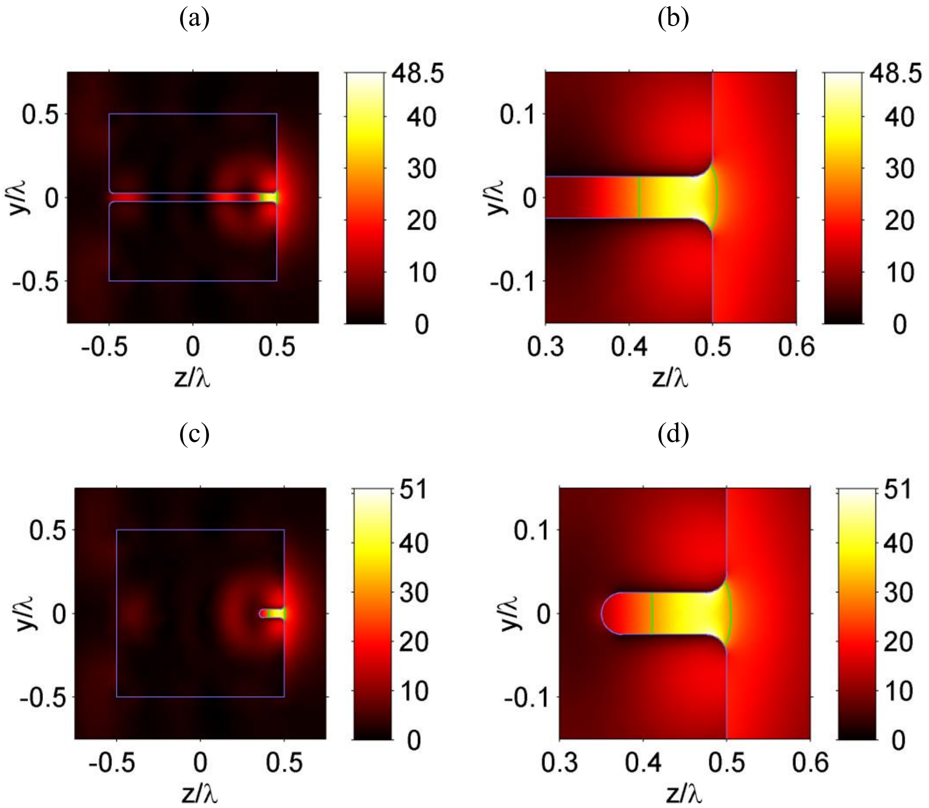

Fig S2. Comparison of the light spot between (a,b) the cuboid with a penetrating nanohole and (c,d) the cuboid with a blind nanohole. It is assumed that the cuboid has edge length of L = λ, the diameter of the nanohole is λ/20, the length of the blind nanohole is 3*λ/20. The refractive index of the cuboid is $n = 2$.
